# Editorial: Microbial C1 Metabolism and Biotechnology

**DOI:** 10.3389/fmicb.2021.744030

**Published:** 2021-08-27

**Authors:** Wei Xiong, Marina G. Kalyuzhnaya, Calvin A. Henard

**Affiliations:** ^1^National Renewable Energy Laboratory, Biosciences Center, Golden, CO, United States; ^2^Department of Biology, San Diego State University, San Diego, CA, United States; ^3^Department of Biological Sciences and BioDiscovery Institute, University of North Texas, Denton, TX, United States

**Keywords:** greenhouse gas, one-carbon metabolism, autotroph, methanotroph, methylotroph, carboxydotroph, formatotroph, biocatalyst

Archaea and bacteria with the capacity to utilize one-carbon (C1) molecules as carbon and energy sources are widespread across the planet and are important players in many biogeochemical processes. As such, they occupy an array of diverse anaerobic and aerobic niches and perform unique metabolisms that enable the utilization of carbon dioxide (CO_2_), carbon monoxide (CO), formate (HCOOH), methanol (CH_3_OH), and methane (CH_4_) for growth. Several industrial processes have been developed that leverage these microbes for the targeted conversion of anthropogenic waste gases that contain C1 molecules, including natural gas (CH_4_), anaerobic digestion-derived biogas (CH_4_ and CO_2_), industrial flue gas (CO_2_ and CO), and syngas (CO_2_, CO, and H_2_) (Figure 1). Further, there is increasing interest in electrochemical reduction of CO_2_ to CO, CH_4_, and intermediates with increased solubility compared to gaseous C1 molecules (formate and methanol) using electrons derived from renewable sources like wind, solar, or hydrothermal systems to mitigate atmospheric greenhouse gas. Thus, C1-utilizing microbes will likely play an integral role in biotechnologies that are part of a sustainable, circular bioeconomy. However, fundamental knowledge gaps into the metabolism and physiology of these microbes exist that limit their utility as biocatalysts for carbon-efficient biomanufacturing. This special topic presents studies focused on the fundamental aspects of C1 metabolism in diverse microbial systems with the ability to convert anthropogenic greenhouse gases into valuable products.

**Figure 1:**
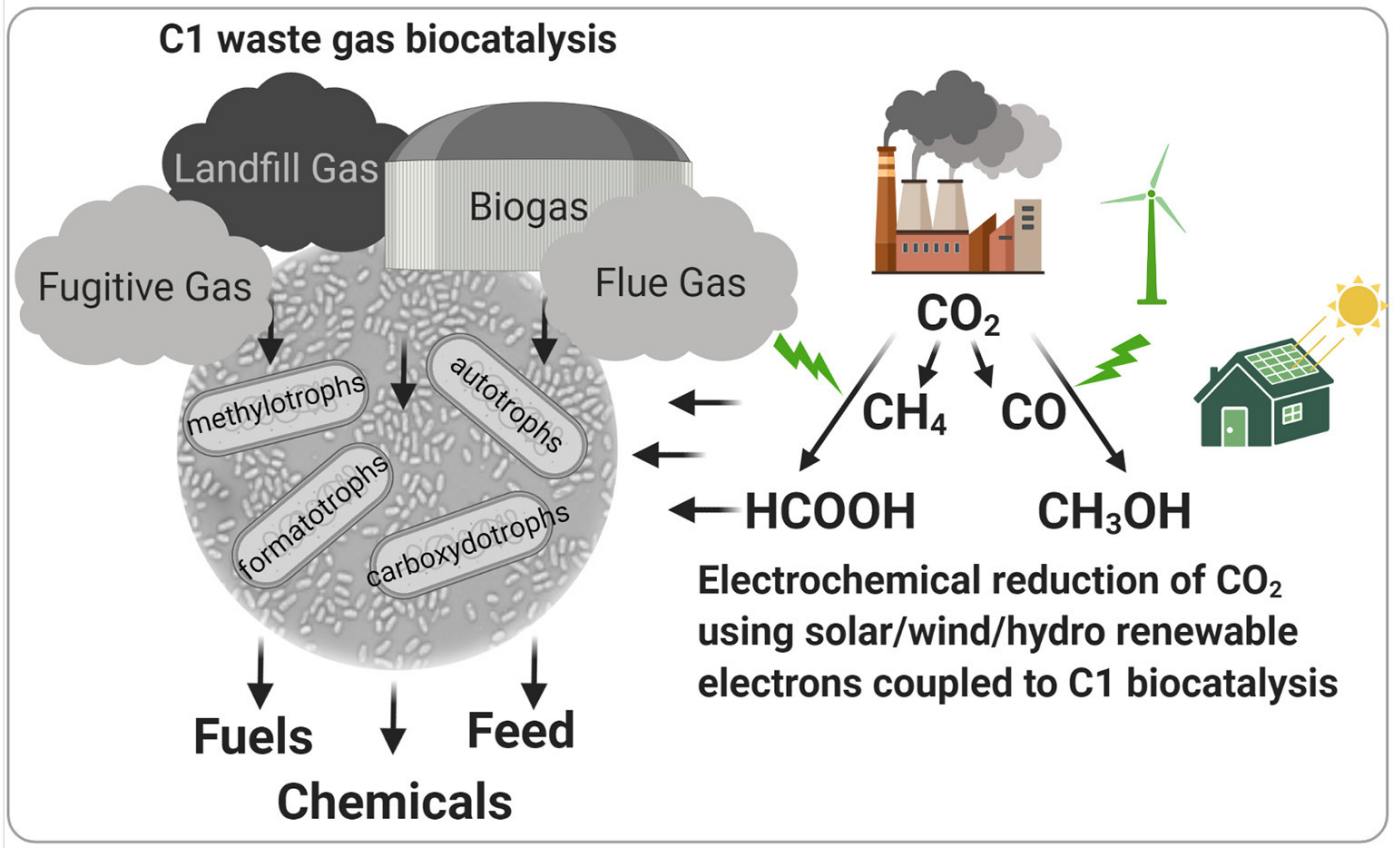
Biotechnologies using C1-utilizing microbes for the conversion of greenhouse and waste gases to valuable products. Specialized microorganisms can valorize one-carbon (C1) sources present in waste gases to fuels, petroleum-derived chemical replacements, and animal and human feed. Electrochemical reduction of industrial flue gas and atmospheric CO_2_ using renewable energy-derived electrons represents a novel, sustainable technology to produce C1 carbon sources for natural or engineered C1 biocatalysts. Figure was created with Biorender.com.

Carbon and energy metabolism are intimately linked in C1-utilizing microbes. For example, CO and CH_3_OH oxidation can be linked to nitrogen and sulfate reduction. Understanding this interaction in more detail may guide future development of microbial processes in new application scenarios such as bioremediation. Alves et al. chose this unique perspective, and their study demonstrates the relationship between CO utilization, hydrogen production, and sulfate reduction in the sulfate-reducing bacterium (SRB) *Desulfofundulus*. Their research further supports the ability of *Desulfofundulus* species to use CO, which is a significant advance. Additional studies focused on CO/syngas fermentation processes for these SRBs, both in sulfate-rich and low-sulfate environments could advance their biotechnological potential (Alves et al.).

While many methane-consuming bacteria can consume CH_3_OH, acetate, or even hydrogen and CO_2_, most of them fail to utilize formate for a yet unknown metabolic bottleneck. Considering that formate is one of the key intermediates of central metabolic pathways, the lack of formatotrophic ability by methanotrophs has remained a puzzling observation for decades. The study by Carere et al. uncovers the physiological mechanism of formate utilization in Methylacidiphilum RTK17.1, a clade of metabolically flexible thermoacidophilic Verrucomicrobial methanotrophs. The authors found a connection between cellular ability to maintain pH homeostasis and its ability to utilize formate for growth. It remains to be seen how widespread the phenomenon is across other clades of methanotrophy (Carere et al.).

Flue gas is a novel, ready-to-use source of carbon that can be converted into chemicals and fuels by acetogenic *Clostridia*. While already proven at an industrial scale, the technology awaits additional advances in fermentation strategies when applied for alternative syngas sources, such as biomass. The study by Ruckel et al. investigates the impacts of trace components of syngas from gasification of biogenic residues on the growth of *Clostridium carboxidivorans*. While some components (H_2_S and NH_3_) in bio-derived syngas stimulate microbial growth, improve CO consumption and alcohol production, other impurities, mainly nitrogen oxides, have severe inhibitory impacts. The study highlights the importance of a thorough investigation of individual and combined effects of trace gases for sustainable technology development (Rückel et al.).

To establish industrially applicable C1 bioconversion process, biosynthetic potential of microbial C1 metabolism needs to be explored comprehensively. For this purpose, researchers desire robust microbial chasses, and the ideal hosts must demonstrate not only C1 utilization, but also the potential for the synthesis of target product in high yield. Particularly, high tolerance to the toxicity of C1 substrates and biosynthetic intermediates could be a key to success. Haupka et al. provided a case study under this topic. They selected a methylotrophic *Bacillus methanolicus* which is genetically editable and can utilize methanol as the sole carbon source for growth and production. They focus on 5-aminovalerate (5AVA), a non-proteinogenic ω-amino acid with potential applications in bioplastics industry. In this study, a state-of-the-art systems biology approach was utilized to identify potential target genes that are responsive to 5AVA. This work elucidated the mechanism of how methylotrophic *B. methanolicus* resists 5AVA toxicity. This study will guide future microbial design for 5AVA production from C1 substrates. Notably, 5AVA production from renewable C1 resources can support the development of novel classes of bioplastics, including, for example, nylon-5,5 (a co-polymer of cadaverine and glutarate) and nylon-5 [an 5AVA homo-polymer proposed as a substitute for petroleum-derived nylon-4,6], as well as various other polyesters (Haupka et al.).

Insights into the evolution of methylotrophic metabolism will guide the development of improved natural or synthetic methylotrophic biocatalysts. *Bacillus methanolicus* displays plasmid-dependent methylotrophy, which is incompletely understood. Schultenkämper et al. compared structure-function relationships between the chromosome- and plasmid-encoded fructose-1,6-bisphosphate aldolase (Fba) variants in the methylotroph *Bacillus methanolicus*. The authors identified amino acid residues that dictate substrate specificity and mediate either glycolytic or gluconeogenic flux by the Fba variants. This study provides insight into the genetic determinants of methylotrophy and identify putative metabolic engineering targets for improved carbon utilization and flux in *Bacillus methanolicus* (Schultenkämper et al.).

C1 biocatalysis represents a promising technology to utilize abundant, squandered C1 carbon sources and mitigate anthropogenic greenhouse gas production. Further, the utilization of renewable energy for the reduction of atmospheric CO_2_ to more reduced C1 carbon sources represents a novel, sustainable route to simultaneously utilize renewable energy and capture and valorize greenhouse gas using C1-utilizing microorganisms. Although several C1 conversion processes have been proven at scale, additional research is required to optimize processes and advance biotechnologies that support a sustainable bioeconomy.

## Author Contributions

CH, MK, and WX wrote the manuscript. The final draft of the manuscript was finalized and approved for publication by all the authors.

## Conflict of Interest

The authors declare that the research was conducted in the absence of any commercial or financial relationships that could be construed as a potential conflict of interest.

## Publisher's Note

All claims expressed in this article are solely those of the authors and do not necessarily represent those of their affiliated organizations, or those of the publisher, the editors and the reviewers. Any product that may be evaluated in this article, or claim that may be made by its manufacturer, is not guaranteed or endorsed by the publisher.

